# Automated Machine-Learning-Driven Analysis of Microplastics by TGA-FTIR for Enhanced Identification and Quantification

**DOI:** 10.1021/acs.analchem.4c06775

**Published:** 2025-04-16

**Authors:** Daniel Prezgot, Maohui Chen, Yingshu Leng, Liliana Gaburici, Shan Zou

**Affiliations:** † Metrology Research Centre, National Research Council Canada, 100 Sussex Drive, Ottawa, Ontario K1A 0R6, Canada; ‡ Ottawa−Carleton Institute for Biomedical Engineering, University of Ottawa, 161 Louis Pasteur, Ottawa, Ontario K1N 6N5, Canada; § Quantum and Nanotechnologies Research Centre, National Research Council Canada, 100 Sussex Drive, Ottawa, Ontario K1A 0R6, Canada

## Abstract

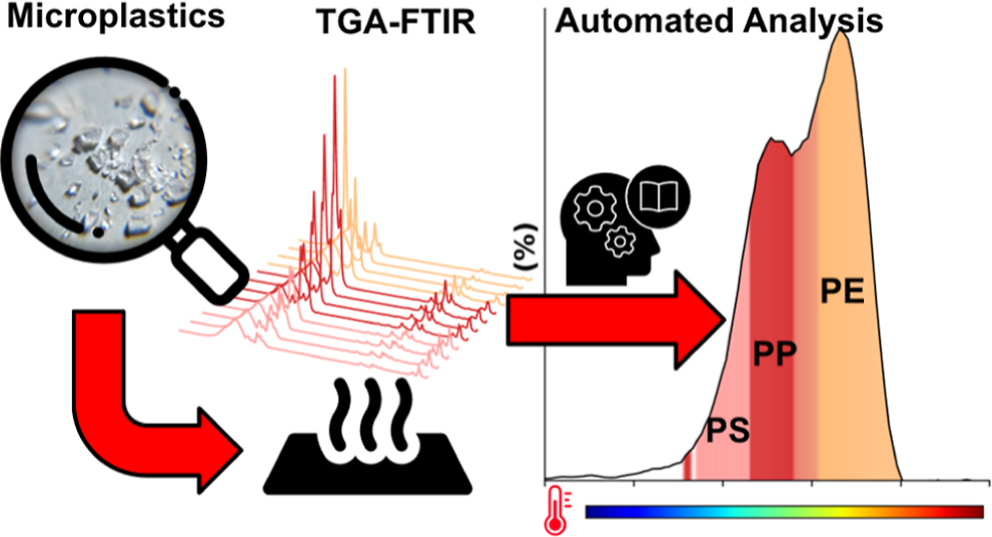

Microplastics persist as ubiquitous environmental contaminants, and efficient methods to quantify and identify their presence are essential for assessing their environmental and health impacts. Common identification approaches typically fall under either vibrational spectroscopy or thermoanalytical techniques with thermogravimetric analysis (TGA) coupled with Fourier transform infrared (FTIR) spectroscopy bridging the intersection. Despite its potential, TGA-FTIR remains relatively underutilized for microplastic analysis, even though each thermogram is associated with approximately 200 FTIR spectra that can be rapidly assessed with targeted automated data analysis. This work explores the development of data analysis routines specialized in identifying plastic components from TGA-FTIR. A dedicated spectral library and a matching algorithm were created to identify polymers from their gas-phase FTIR spectra. The approach was further enhanced by utilizing machine learning (ML) classification techniques, including k-nearest neighbor, random forest, support vector classifier, and multilayer perceptron. The performance of these classifiers for complex data sets was evaluated using synthetic data sets generated from the spectral library. ML techniques offered precise and unambiguous identification compared with a custom spectral matching algorithm. By correlating polymer identities with mass loss in the thermogram, this approach combines qualitative insights with semiquantitative analysis, enabling a streamlined assessment of plastic content in samples.

## Introduction

Since the large-scale production of plastics began in the 1950s, over 7000 Mt of plastic waste has cumulatively been generated, with rates of plastic production and thus waste still projected to increase.^1,2^ This waste includes primary and secondary microplastics formed through degradation, along with smaller nanoplastics.^3^ These microplastics are pervasive and found in air, water sources, soil, and even food.^3^ As microplastics accumulate in the environment, they pose potential ecological and toxicological hazards.^4,5^ It is of significant interest to identify and quantify environmental plastics in order to assess and monitor their prevalence, persistence, and impact.

A myriad of methods have been deployed for the characterization of micro(nano) plastics;^6,7^ however, only a small subset specifically focuses on identifying the polymer constituents in the environmental plastics. Among these, the leading techniques include vibrational spectroscopy, thermoanalytical methods, and mass spectrometry-based techniques.^8^ Vibrational spectroscopies, namely, Raman spectroscopy and Fourier transform infrared (FTIR) spectroscopy, identify plastics by the unique fingerprints in scattering or absorption bands resulting from molecular vibrations. These techniques can also be combined with microscopy to assess the size and morphology of the particles examined.^8^ Alternatively, mass spectrometry-based techniques, such as pyrolysis gas chromatography/mass spectrometry (Py-GC/MS),^9^ thermoextraction and desorption (TED-GC/MS),^10^ and matrix-assisted laser desorption/ionization time-of-flight mass spectrometry (MALDI-TOF MS),^11^ offer high sensitivity and precision in identifying and quantifying polymer types. In contrast, thermoanalytical methods, such as thermogravimetric analysis (TGA) and differential scanning calorimetry (DSC), utilize the thermal behavior of polymers to distinguish them. DSC measures heat flow associated with specific thermal transitions, such as melting, crystallization, and glass transition temperatures, offering detailed information on the polymer’s phase behavior and thermal properties. TGA focuses on the thermal decomposition of polymer products by measuring the weight loss as a function of temperature. The gases evolved during the TGA process can be readily analyzed by other techniques, such as mass spectrometry and FTIR, providing an alternative comprehensive, low-complexity thermoanalytical technique.^12^

TGA-FTIR is capable of identifying polymers spiked into a sample with accuracy comparable to other, more complex thermoanalytical techniques.^12,13^ It has been applied to the analysis of microplastics in marine organisms, water, and soil.^14,15^ Despite these demonstrations, this technique remains an underutilized tool in the analysis of microplastics.^16^ TGA-FTIR provides a greater depth of information compared with room-temperature IR (RT IR), as FTIR spectra are continuously monitored during the polymer decomposition process. This generates hundreds of spectra that can be correlated with temperature to provide insights into polymer identities, additives, and decomposition behavior.

With such a rich data set, chemometric approaches are required for matching the spectroscopic data to specific polymers. Vibrational spectroscopic analysis, for instance, often relies on comparing spectra to a library of known references using metrics such as Euclidean distance, Pearson correlation, or more sophisticated algorithms.^17−19^ However, these methods can fall short when applied to environmental samples, where matrices, mixtures, and additives complicate the spectra. Environmental factors, polymer aging, and the presence of additives or modified products can significantly alter spectroscopic signatures, making them difficult to match to standard spectral libraries. In these cases, gas-phase infrared spectroscopy can provide additional insights, but it still faces challenges in handling complex mixtures and altered polymers. Elevated temperatures can help separate additives and reduce matrix interferences (e.g., cellulose), making techniques such as TGA particularly useful. TGA isolates and characterizes different components by their thermal decomposition profiles, aiding identification when traditional spectroscopic methods prove to be insufficient.

By integrating advanced data analysis of the FTIR spectra with the inherent ability of TGA to isolate polymers based on their unique thermal properties, samples can be rapidly assessed for plastic content. Furthermore, machine learning (ML) is an emerging tool in chemometrics, capable of extracting intricate features that may otherwise not be obvious.^19−21^ Numerous recent studies demonstrated the automation of plastic identification by vibrational spectroscopy with high accuracy, even in challenging scenarios involving complex mixtures or modified polymers.^22−25^ Machine learning techniques, such as support vector classifiers (SVC),^22^ k-nearest neighbors (kNN),^26^ random forest classifiers (RF),^27^ and deep learning models,^28^ have all been applied to plastics identification from vibrational spectra.^29^ These methods have surged in popularity due to their accessibility through open source libraries, and modern computing power, as well as for their potential in terms of accuracy, speed, and interpretability.

This work investigates the application of chemometric techniques to automatically identify and quantify plastic constituents in samples analyzed by TGA-FTIR. First, a library of TGA-FTIR data is constructed, consisting of the most commonly produced sources of environmental contamination. This is necessary as existing FTIR databases for polymers typically include only room-temperature spectra, which differ from the gas-evolved products measured in TGA-FTIR and cannot always be relied upon for accurate identification.^30^ By using a more complete and appropriate data set, custom data analysis routines leveraging Pearson correlation or machine learning classifiers optimized for this problem were developed. These methods were evaluated on a large set of synthetic data sets and real microplastic mixtures to evaluate their ability to detect the presence and reveal the identities of plastics in increasingly complex samples. Secondarily, the prospects of combining the identification results with TGA for semiquantitative analysis was investigated.

## Experimental Section

### Materials

Polymer materials used for building the reference library were sourced from various manufacturers and provided in the form of pellets or powders, as detailed in Table S1.

### Thermogravimetric Analysis Coupled with FTIR

Thermogravimetric analysis was carried out using a Netzch TG 209F1 Iris (TGA-MS-FTIR) system. Samples of 5–20 mg were loaded into an aluminum oxide crucible and heated from 40 to 1000 °C at a rate of 10 °C/min in an argon atmosphere, with a total run time of under 2 h. The TGA was coupled to an FTIR spectrometer (Bruker Tensor 207, Opus 8.5 software) via a 1.5 m transfer line maintained at 200 °C, with the dwell time in the transfer line being approximately 2.5 s. Thermogravimetric (TG) curves were generated and subsequently used to calculate differential thermogravimetric (DTG) curves. Blank measurements of an empty crucible were used to correct TG curves and to provide reference FTIR spectra.

### Data Processing

Data processing was performed using an Anaconda Python (v3.11) distribution utilizing the Spyder IDE and Jupyter Notebooks on an Intel i5-1145G7 CPU with 16 GB of RAM. Machine learning models were built using scikit-learn (v1.40).

FTIR spectra from each TGA run were baseline-corrected using adaptive smoothness penalized least-squares algorithm^31^ via the pybaselines package.^32^ The TG data was interpolated to match the exact temperature points of the FTIR spectra. To account for the temperature offset introduced by the transfer line dwell time, the recorded temperatures of the FTIR spectra were adjusted accordingly. Only spectra within the 800–4000 cm^–1^ range and the TG and FTIR data were taken between 150 and 750 °C, resulting in approximately 250 FTIR spectra in each TGA run (data set). Savitzky–Golay smoothing was applied to the DTG data prior to plotting.

The CO_2_ region and OH regions, 2200–2400 cm^–1^ and 3150–3500 cm^–1^, respectively, were excluded from each spectrum due to sample matrix or atmospheric leakage, which rendered these sections uninformative. Spectra were then normalized by standard normal variate (SNV) prior to spectral matching or inclusion in training data sets. Feature selection was employed using an ANOVA f-test to pick out the 200 most covariant data points across the library (Figure S2) to reduce the dimensionality of training data to prevent overfitting or poor model performance.^33^ The temperature corresponding to each spectrum was included as a feature, and SNV scaling was again applied to the training data set.

### Identification by Spectral Matching

The FTIR spectra that constitute the library were incorporated into a customized spectral matching algorithm (SMA). Hit quality (*r*) is calculated for each spectrum in the TGA-FTIR data set against every reference FTIR spectrum in the library. This metric combined the average Pearson correlation of the original/raw spectra (*r*_0_) and their respective first derivatives (*r*_1_), with the latter improving sensitivity by minimizing baseline shift. Pearson correlation was chosen over the industry-standard Euclidean distance metric^18^ for its higher sensitivity.^34^ A threshold of *r* > 0.7 was defined for positive matches based on empirical testing and literature values.^35^ Thermal data are incorporated into the SMA as a single-point penalization term to adjust *r* values based on temperature differences between each data spectrum (*T*_data_) and each reference spectrum in the library (*T*_lib_)
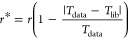
1

If a match exceeded the threshold (*r* > 0.7), the residual spectra were re-evaluated to identify additional components. This iterative approach allowed for multiple classifications in mixtures (Figure S1).

### Machine Learning Model Training

Four machine learning classifiers were used: k-nearest neighbors (kNN), multilayer perceptron (MLP), RF, and SVC. Classifiers were trained to predict polymer identity based on an assembled TGA-FTIR library, as it cannot be easily predicted which model will exhibit the best performance beforehand. A one-vs-rest strategy was used as a consistent multiclass methodology, fitting a binary classifier for each class (plastic), e.g., polyethylene vs all others. Labels were binarized to enable multilabel identification so that more than one class could potentially be identified in any given spectrum.

Training of the models was done using standard train/test/split methodology, where all available spectra were split 75%/25% between training and testing data using the code adapted from Lei et al.^23^ Hyperparameter tuning was performed for each model using a grid search methodology with 3-fold cross-validation (Table S2).

The outputs of the machine learning models were probabilistic to be more directly comparable to those of the spectral matching method and to apply a threshold to reduce false positives. Each model outputted the probability of any given spectrum being any given polymer in the library, and a threshold was implemented, below which no class was confirmed as a positive match.

## Results and Discussion

### Assembly of the TGA-FTIR Library

A baseline library of TGA-FTIR data was essential for accurately predicting the constituent polymers in a sample. Most existing open-source or commercial libraries dedicated to polymers include only room-temperature FTIR data, which can greatly differ from the volatile decomposition products observed in the TGA-FTIR analysis. Room-temperature FTIR spectra do not necessarily capture the structural and compositional changes observed in the volatile decomposition products of polymers at elevated temperatures, which are critical for understanding their complete decomposition profiles (Figure S3). Thus, a specialized library of TGA-FTIR data was developed to address this need.

Construction of this library began with the collection of full thermograms and corresponding FTIR data for ten types of the most commonly produced plastics, which are most frequently found in environmental samples. These plastics were each sourced from 2 to 3 different suppliers, including polyethylene (PE), polypropylene (PP), polystyrene (PS), polyethylene terephthalate (PET), polyamide (PA), polyvinyl chloride (PVC), polyurethane (PUR), poly(methyl methacrylate) (PMMA), polytetrafluoroethylene (PTFE), and polycarbonate (PC). Multiple (2–3) samples of each polymer were included to account for variations in properties such as molecular weights, physical form (e.g., powders, fibers, or pellets), and molecular structure (e.g., polyamide 6 and 6-6). However, the representation of the polymers was not exhaustive or truly representative of environmentally sourced plastics. Expanding the number of sources could improve the applicability and robustness of the technique. Cellulose (CEL) was also included in the library as a common environmental matrix component, allowing it to be distinguished from the synthetic polymers of interest.

**Figure 1:**
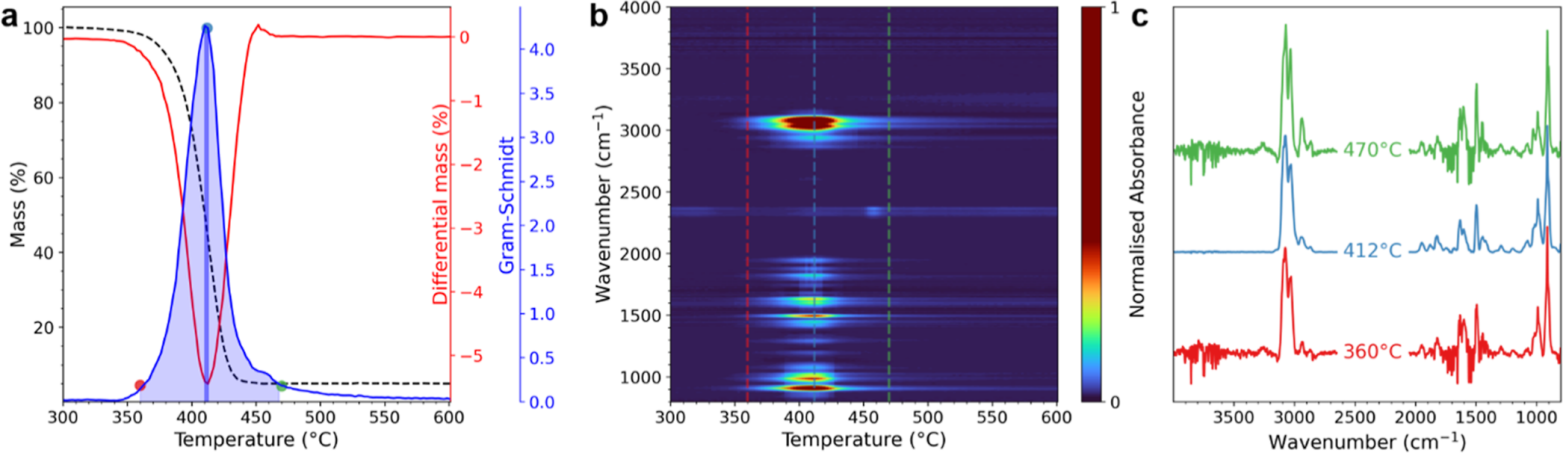
(a) Thermogram (dotted line), differential thermogram (red solid line), and Gram–Schmidt (integrated FTIR absorbance; blue solid line) of a polymer included in the library, in this case polystyrene. (b) FTIR absorbance map across the temperature range, highlighting prominent features in the fingerprint (<1800 cm^–1^) and the CH region (∼3000 cm^–1^). The dotted lines denote the ROI and the maximal absorbance. (c) Corresponding spectra from (b) contrasting the clarity of spectral features.

To ensure the relevance and accuracy of the data, only spectra within a defined region of interest (ROI; Table S1) were incorporated into the library for spectral matching or ML training data. The temperature ranges were selected to capture the most significant decomposition events, accurately representing the polymer’s behavior during pyrolysis (Figure 1a–c). For polymers with multiple DTG peaks, such as PMMA and PVC, spectra from each peak were used to provide a comprehensive representation of their thermal degradation profiles. The final library was assembled from 27 samples and contains a total of 2815 FTIR spectra. With the library assembled, a method is required to accurately identify polymers from these spectra. While the TGA process inherently provides some degree of separation between polymers that decompose at different temperatures, many thermograms overlap between 350 and 450 °C, yielding in FTIR spectra containing mixed components. Only a few of the polymers used in this work, such as PMMA and PTFE, showed maximum decomposition temperatures outside this range. This can interfere with spectral identification, as secondary components may be difficult to discern from a single spectrum.

**Figure 2:**
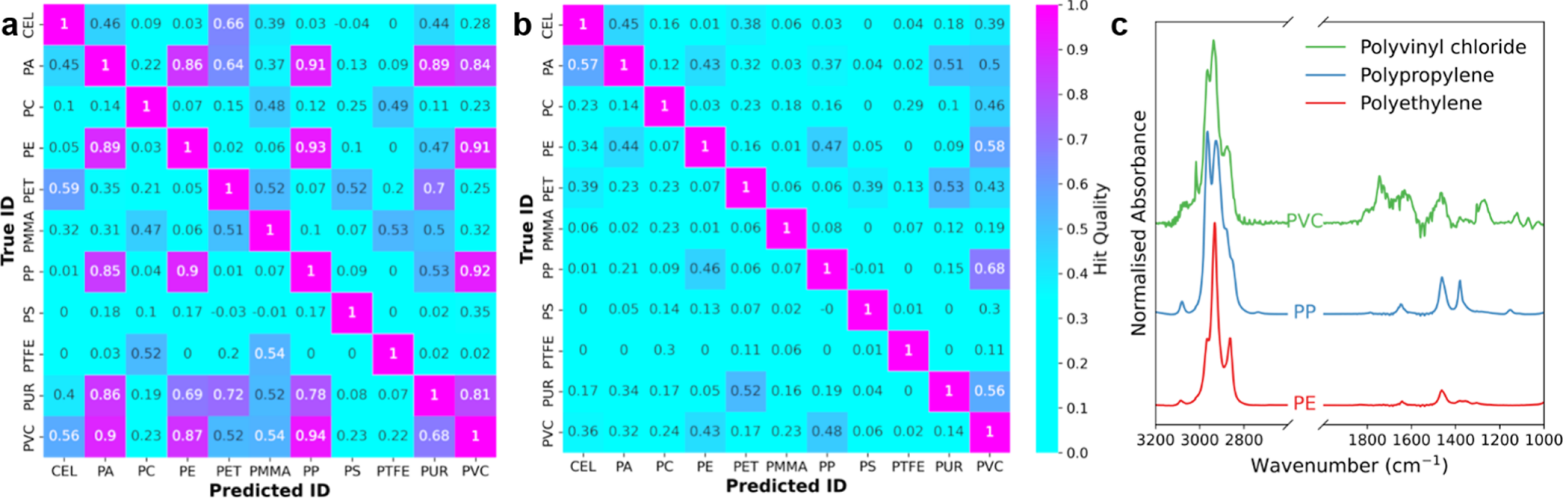
(a) Pearson correlation matrix indicating the maximum correlation (*r*) values between every polymer spectrum in the library. High values outside the diagonal indicate polymers with an inherent similarity in their spectra. (b) The same correlation matrix but applied on the derivative spectra. This improves the distinction between samples. (c) Gas-phase IR spectra produced for PE, PP, and PVC. The Pearson correlations between these spectra are >0.9 with respect to each other; too similar to be distinguished using Pearson correlation alone.

Further compounding the issue, gas-phase FTIR spectra of some polymers have inherently high similarity. Figure 2a displays a Pearson correlation matrix of the matching process run on the library itself. Numbers outside the diagonal represent the spectral similarity between different polymers. For example, when a library spectrum contains PE, PP is given an *r* value of 0.93: enough to be considered a positive match given a threshold of 0.7.^35^ Other sources of confusion can occur between polymer groups such as PET and PUR due to similar functional groups and decomposition products in their TGA-FTIR spectra. When using pearson correlation, these errors can be mitigated by matching against the derivative spectra (Figure 2b), allowing for discrimination between similar spectra such as those shown in Figure 2c. Matrix effects, impurities, and contributions from other polymers in the mixture can further complicate spectral identification. Techniques that enable decisive discrimination are essential to simplify the interpretation of results, reduce ambiguous matching, and lessen the burden on the user performing the analysis.

### Identification by Machine Learning

Machine learning (ML) techniques have become an increasingly popular tool for spectral identification due to their potential advantages in rapidity and accuracy over conventional spectral matching techniques. TGA-FTIR data sets naturally lend themselves to ML by providing dense, feature-rich training data. A number of models were trained on TGA-FTIR data including kNN, MLP, RF, and SVC. Multiple models were tested, as it is difficult to predict which ML model will perform best for a given problem. Additionally, a custom SMA was used, which improves upon the use of Pearson correlation by utilizing the first derivative of each spectrum and its respective temperature to alter the final *r* value as outlined in the Experimental Section (Figure S4a).

Training data was assembled using FTIR data previously obtained from ten types of plastics (25 samples) and two cellulose samples (Table S1). Incorporating data from various plastics as well as cellulose across a broad temperature range exposes the models to diverse scenarios, enhancing the ability to distinguish between materials. The temperature of each spectrum is included as a feature, although it is unclear to what extent this influences the models.

FTIR data in each data set were chosen based on the ROI where identifiable features were observed in the DTG and Gram–Schmidt (integrated FTIR) profiles of the thermogram (Table S1). By focusing on these ROIs, the model is trained on the most relevant data, which helps avoid confusion from the less informative regions of the spectra. Excluding regions without significant features prevents the model from learning irrelevant or noisy information that does not contribute to the classification of the spectra. To correctly identify true negatives, spectra outside of the ROIs were included in the training data as well as plastic-free blank samples. This approach yielded approximately 45–100 FTIR spectra for each TGA-FTIR data set in the model, depending on the width of the DTG curve(s). The data set was augmented using the extended multiplicative scatter augmentation (EMSA) technique,^36^ which artificially introduces small fluctuations in the spectrum, further extending the data set with spectra containing slight differences from the experimental data. EMSA was used to generate 300 spectra for each class (including blanks) for a total of 3600 new entries. It was verified to not negatively impact cross-validation scores during model testing and yielded a slight increase in accuracy during model validation. A full description of augmentation process is provided along Figure S5 in the Supporting Information.

**Figure 3:**
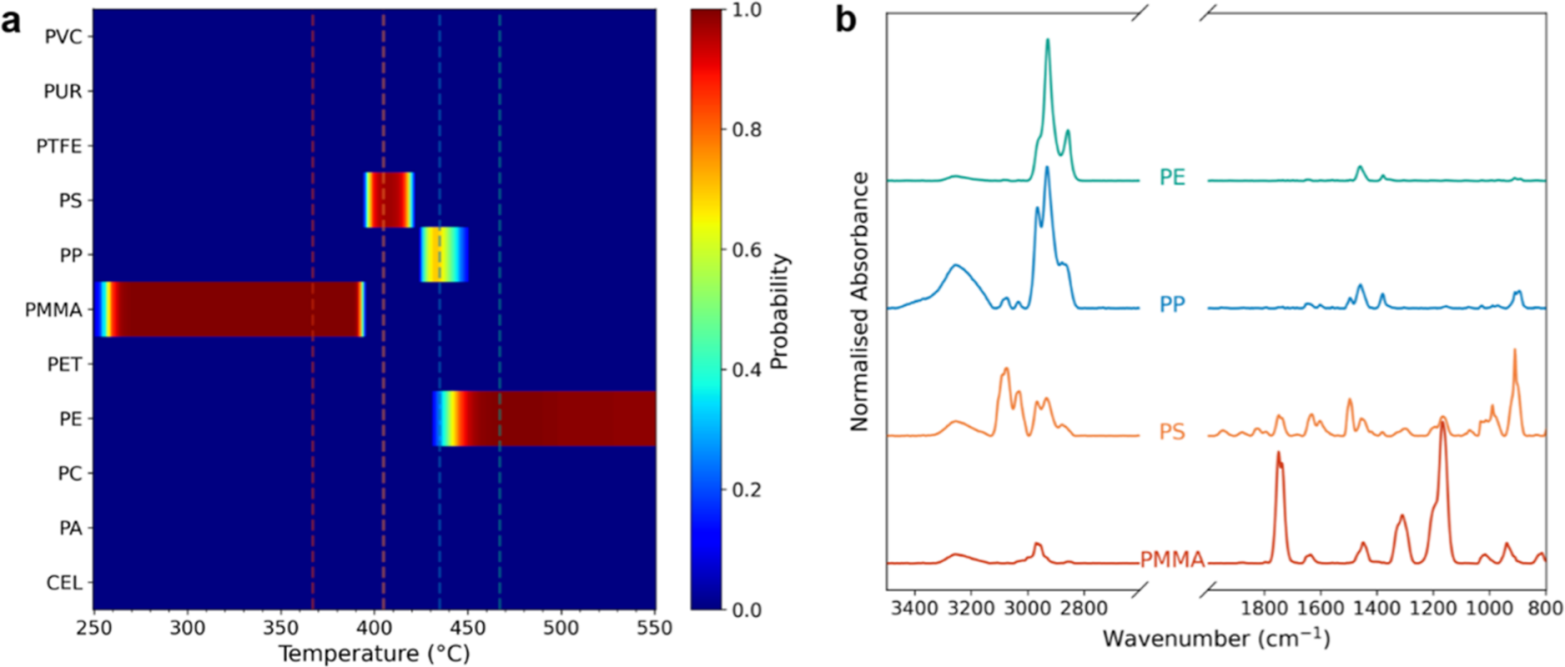
(a) Probability heatmap showing the results of machine-learning-based classification by SVC for TGA-FTIR analysis of a sample containing a mixture of PMMA, PS, PE, and PP powders. (b) FTIR spectra attributed to the components of the mixture at the temperatures denoted by the dashed lines in (a).

The models output a list of probabilities of each polymer with respect to temperature in the thermogram, represented as a heatmap in Figure 3a. The models tended to produce decisive results, with probabilities often either well above the established threshold or close to zero. This is due to the nature of probability calculations in the models: for example, in the MLP, the final classification is performed by fitting to a logistic sigmoidal activation function in the output layer; thus, the final value favors the extremities of the curve (toward 0 or 1) and is less likely to be found toward the center (0.5). Areas where the probability is lower can be due to lower signal-to-noise or the presence of peaks due to other components in the mixture, such as how some signals from PS and PE are visible in the PP spectrum (Figure 3b). This is in contrast to the SMA, which can display relatively high hit qualities for items that are not actually in the sample (Figure S4b).

### Evaluating Model Performance

In the case of single-label (samples containing only one polymer) data, the accuracy of all machine learning models was exceptional, being close to 1 during model training. While models had high accuracy during training and testing, they displayed relative insensitivity to hyperparameters, indicating a potential for overfitting. However, this evaluation is also misleading, as real-world samples often contain multiple polymers. Thus, it is necessary to evaluate how the models will perform on samples that contain mixtures of polymers.

**Figure 4:**
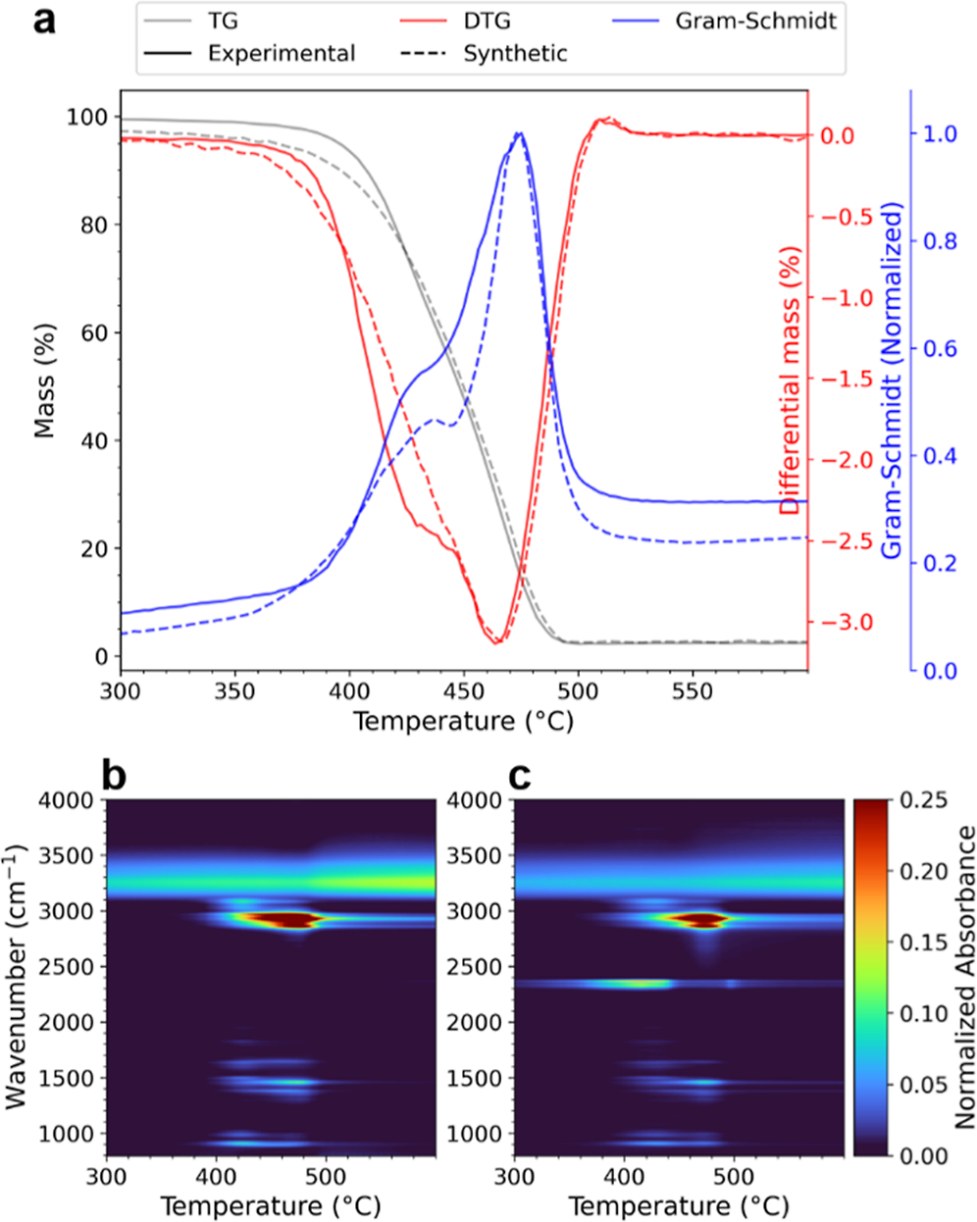
(a) TGA-FTIR data set showing the thermogram, DTG, and integrated FTIR absorbance (Gram–Schmidt) profile and for a mixture of powdered PS, PP, and PE, as well as a synthetic data set made to resemble the same mixture. A full map of the FTIR data is shown in (b) and (c) for the experimental and synthetic data, respectively. The only key difference in FTIR data is the CO_2_ and OH bands, which are ignored features. Both data sets show very similar thermal and FTIR profiles, demonstrating the validity of the synthetic TGA-FTIR data.

It would be impractical to experimentally produce a sufficient number of plastic mixtures that represent the potential combinations of polymers required to properly test the model. To this end, a synthetic data set was created, which randomly combined TGA-FTIR data sets from 2 to 4 different polymers in randomized fractions, creating a final data set with 1028 thermograms containing 554 unique combinations. The synthetic data mixtures were found to resemble experimentally measured mixtures (Figure 4a,b). Differences between the experimental and synthetic data could be found in the overall infrared absorbance, which is randomized in the synthetic set, and shifts of decomposition temperatures sometimes observed in experimental mixtures.

Table 1 summarizes the performance of the tested models on the synthetic data set. The ground truths were established by the identities present in each thermogram rather than at the level of individual spectra, from which the model performs identification. Recall, or sensitivity, refers to the ability of a model to return true positive labels, whereas precision represents the ability not to label false positives. *F*_1_-score is the harmonized average of precision and recall, representing the overall performance, taken as the macro average *per class*. Accuracy was defined by the Hamming accuracy score, representing the average fraction of correct classes determined *per sample*. This best represents the practical accuracy of the model; in any given sample, what fraction of classes will be predicted correctly? Most models showed a preference toward high precision, while suffering a relatively weak recall, with this trend being somewhat reversed in the case of the kNN model and the SMA.

**Table 1:** Model Performance Metrics Evaluated on a Synthetic Multilabel Data Set

classifier	precision	recall	*F*_1_-score	accuracy
kNN	0.84	0.90	0.84	0.73
MLP	0.94	0.88	0.90	0.85
RF	0.97	0.80	0.86	0.81
SVC	0.99	0.86	0.91	0.87
SMA	0.91	0.93	0.91	0.83

**Figure 5:**
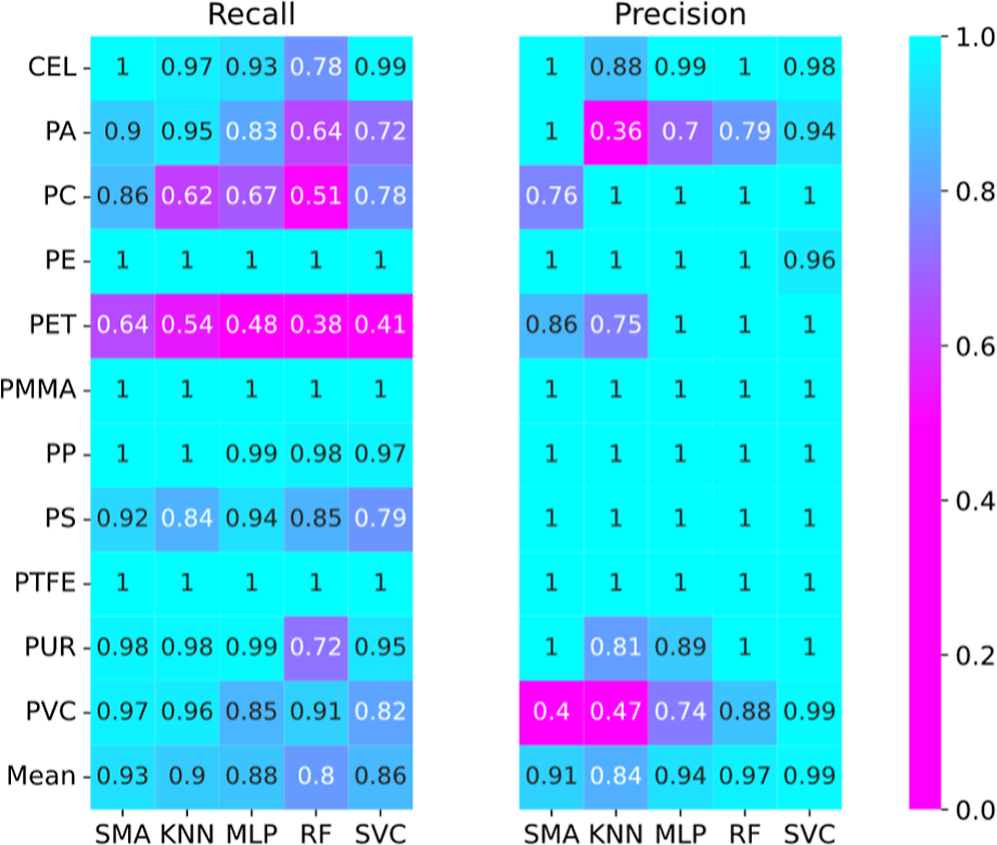
Recall and precision chart of all models using the synthetic data set. Most models generally showed good precision but lower recall. In either case, deficiencies in recall or precision were primarily caused by a limited number of classes.

The best performing classifier was the SVC, with the *F*_1_-score and Hamming accuracy scores of 0.91 and 0.87, respectively, followed closely by the MLP classifier. The previously described SMA performed similarly to the best ML classifiers, having an *F*_1_-score of 0.91 and comparable accuracy, though it was more prone to false positives. The Hamming accuracy of these models was 80–90%, indicating that not all samples were being completely identified. A closer look at the per-class performance metrics in Figure 5 reveals that a select few polymers disproportionately negatively affect the accuracy of the models. In the case of the SVC model, all classes displayed excellent precision, with a mean of 0.99, but some suffered from poor recall, indicating a tendency for not all classes to be correctly identified as being present in a sample. These include PA, PC, PS, and, in particular, PET, which universally was the poorest performing class. There was no indication of this shortcoming in the case of the single-label validation data; thus, these issues only occur in mixtures. This is likely due to the relatively weak bands in the vibrational spectrum for PET, with the exception of the C=O band at 1760 cm^–1^, which is not distinctive to this polymer. Thus, there is a reduced ability to distinguish PET in the presence of other polymers with stronger vibrational modes.

For general applicability, the performance of all classes must be optimized. The performance of the ML models could be further improved by having access to more training data. Additionally, a more suitable feature selection routine that is better suited to spectroscopic data may help improve performance in weakly performing classes.^37^ Alternatively, dimensionality reduction techniques, such as principal component analysis, may be employed to better cluster the data before analysis. The overall metrics of either the SMA or the better performing ML models show promise in identifying plastics in complex mixtures. Lastly, the models, at this stage, are performing a multilabel task based on single-label training data. A more specialized multilabel model may be better suited for this task.

### Qualitative and Quantitative Analysis of Environmental Samples

The complexity of the analysis further increases when applied to environmental microplastics. In addition to the unknown quantities of potentially several different polymer types, environmental weathering can alter their spectroscopic signatures,^38^ and the sample may contain a sample matrix that further interferes with analysis. Utilization of TGA-FTIR alleviates some of these issues as the spectral signature of the volatile products measured by TGA-FTIR is less influenced by weathering (Figure S6), and the thermal gradient can separate out both the nonvolatile and easily pyrolyzed matrix components.

**Figure 6:**
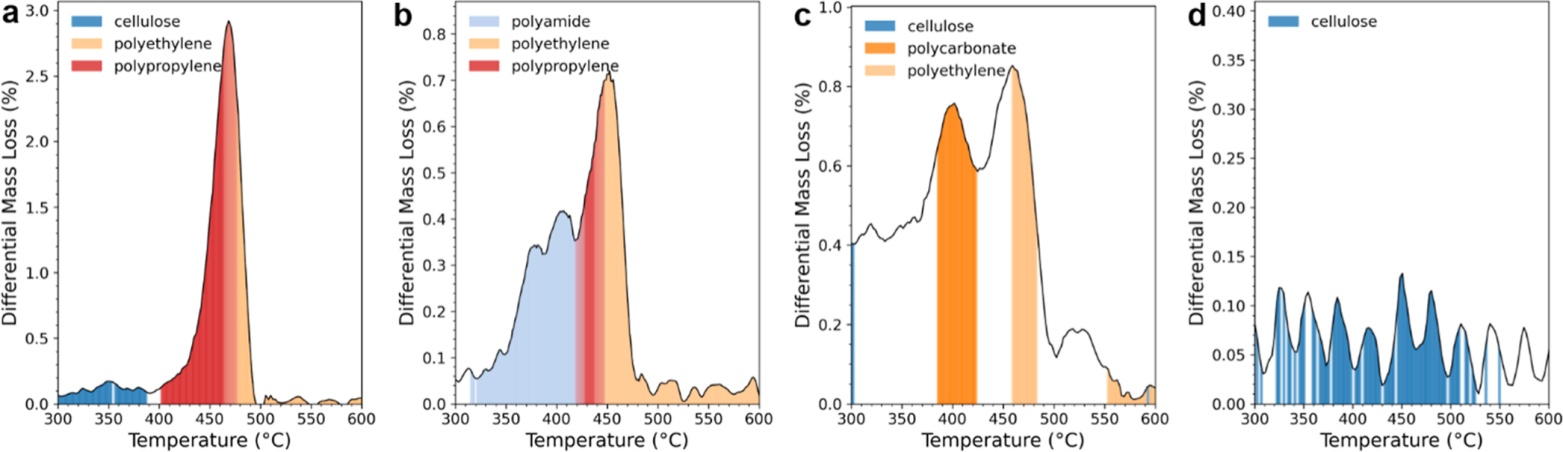
DTG curves overlaid with the assignment of each part of the thermogram performed by fitting the SVC model to FTIR data of a municipal biosolid-derived matrix which was spiked with microplastics (a and b). The relative compositions are estimated by summing the mass loss assigned to each identity: (a) PE and PP: 54%, 46%, spiked prior to density separation and (b) PA, PET, PP, and PE: 42%, 0%, 43%, and 15% added to the postseparation matrix. (c) The results of a biosolid-treated soil sample found to contain PC and PE confirm the presence of plastics. (d) Blank biosolid matrix without any added plastics.

In addition to qualitative information, TGA-FTIR has the potential to simultaneously provide quantitative data as the TGA component records the mass of the volatilized components. The mass loss can be correlated to the polymers identified by FTIR, allowing the relative compositions of each component to be estimated by integrating the portion of the DTG curve assigned to each polymer. In cases where multiple components are identified from the spectrum, the contribution of each component in the mixture is estimated by multivariate curve resolution.^39^ To evaluate the chemometric techniques described in this work for identifying and quantifying plastics in environmental samples, samples were prepared by spiking an environmental matrix that was confirmed to contain no detectable plastics. The matrix consisted of municipal wastewater biosolids, which had undergone density separation and filtration.^40^ The recovered solids were spiked with approximately equal amounts of PE and PP (Figure 6a), or PE, PP, PET, and PA (Figure 6b). Figure 6 displays the combined qualitative and quantitative results of the analysis of two of these samples, showing the classification of each polymer by the SVC model overlaid with the DTG curve; the area under this curve represents the mass loss during pyrolysis of each component.

In the aforementioned samples, this routine successfully distinguished PE and PP in a binary mixture, assigning the relative compositions of 54% and 46%, respectively, which agreed with expectations. However, in the quaternary mixture containing PA, PET, PE, and PP, PET was not detected at all, and PP was only sparingly observed and quantified. Each polymer was expected to be present in equal quantities, but the observed compositions were 42%, 0%, 43%, and 15% for PA, PET, PE, and PP, respectively. The models are estimated to be able to identify the presence of polymers in masses down to 0.05–0.5 mg; however, as the number of components in a mixture increases, detection becomes less reliable (Figure S7). PET is particularly problematic to detect in more complex samples (Figure 3). The region from 400 to 420 °C, where the onset and peak decomposition of PET should occur, is instead misclassified as PA. The compositions of PA and PE are higher than expected as temperatures containing mixed spectra are disproportionately misclassified. The sample matrix itself was found to have a negligable impact on classification and quantification; the blank shown in Figure 6d has minimal mass loss and no identified polymers in the region of interest.

The results of the analysis of a biosolid-treated soil sample are displayed in Figure 6c. The sample was prepared in a fashion previously described by density separation and filtration on 50 g of soil,^40^ followed by TGA-FTIR analysis on a 3 mg mass of extract. While large portions of the thermogram remain unclassified due to unknown contributions from the sample matrix or other polymer components, some portions of the data are classified to PC and PE with a high degree of certainty. From the areas identified, it was estimated that PC and PE constituted 12% and 8% of the extracted sample mass, respectively.

The current quantification estimate is limited by not accounting for the latent residual mass of each polymer; the expected mass loss is different for each polymer and would have to be accounted for in a proper quantitative model. Furthermore, when more than one component is identified at any given temperature, the contribution from each spectrum is estimated by MCR, but the fraction of spectral contribution does not necessarily correlate with the mass fraction. Despite the limitations, the degree of quantification is suitable for screening and can provide a rough estimate of the relative plastic content in a given sample.

## Conclusions

The creation of a custom library of TGA-FTIR enabled the data-driven approach presented here, which enables in-depth, rapid, and generalized identification of plastics by TGA-FTIR. Machine learning models, in particular, SVC or MLP classifiers, were capable of accurately identifying polymers present in TGA-FTIR data sets containing a single polymer with nearly 100% accuracy and in samples containing multiple (2–4) polymers with an average classification accuracy approaching 90%. A traditional spectral matching approach demonstrated comparable accuracy and remains a valid method for spectral identification. However, it comes at the cost of a higher rate of false positives, slower analysis time, and more ambiguous interpretation of results. In contrast, machine learning approaches were rapid and computationally efficient and provided unambiguous results. Room for improvement exists, as performance declined with increasing complexity of samples. Models better suited for multilabel classification should be explored. Fortunately, machine learning techniques are scalable, as the expansion of available training data can improve model performance and make it more robust to weathered plastics, additives, and other sources of matrix interference. The potential to extract quantitative data by combining the model classification results with TGA was also evaluated, and while imprecise, it could still be suitable for validation of the classifiers and screening of samples for further analysis. The models in this work were primarily trained on FTIR data, but their scope can be expanded to include and interpret thermogravimetric data and other detection methods such as GC/MS to improve the qualitative and quantitative accuracy of the technique.

## Data Availability

The library TGA-FTIR data sets, code for training and fitting of models, as well as data visualization tools are available on the NRC Digital Repository 10.4224/40003458.

## Supporting Information Available

List of materials used; hyperparameters used in model training; illustration of the residual matching process; feature selection; comparison of room-temperature and gas-phase TG-FTIR spectra; correlation matrix and correlation heatmap; example of augmented spectra; comparison of TGA-FTIR and ATR-FTIR spectra; and evaluation of classification limits (PDF)
