# Supplementary material for: Automated Machine-Learning-Driven Analysis of Microplastics by TGA-FTIR for Enhanced Identification and Quantification
Source: Anal Chem. 2025 Apr 16;97(16):8833–40. doi: 10.1021/acs.analchem.4c06775 (PMC12044591; doi:10.1021/acs.analchem.4c06775)
Supplement: Supplementary file 1 — ac4c06775_si_001.pdf [file ac4c06775_si_001.pdf]

SUPPORTING INFORMATION

Automated Machine-Learning Driven Analysis of Microplastics by TGA-FTIR for Enhanced Identification and Quantification

Daniel Prezgot* [a], Maohui Chen [a], Yingshu Leng [a,b], Liliana Gaburici [c], and Shan Zou* [a]

a Metrology Research Centre, National Research Council Canada, 100 Sussex Drive, Ottawa, Ontario K1A 0R6, Canada

b Ottawa–Carleton Institute for Biomedical Engineering, University of Ottawa, 161 Louis Pasteur, Ottawa, Ontario K1N 6N5, Canada

c Quantum and Nanotechnologies Research Centre, National Research Council Canada, 100 Sussex Drive, Ottawa, Ontario K1A 0R6, Canada

TABLE OF CONTENTS

Item	Description	Page
**Table S1**	Polymers, their sources, and the temperatures included in the polymer library used for this work.	S2
**Figure S1**	Illustration of the residual matching process in the customized spectral matching algorithm	S3
**Figure S2**	Feature selection with n = 200 points, exemplified on a dataset with a mixture of polymers	S3
**Table S2**	Hyperparameters used in model training	S4
**Figure S3**	Comparison of room-temperature and gas-phase TG-FTIR spectra	S4
**Figure S4**	Correlation matrix and correlation heatmap for the custom spectral matching algorithm	S5
**Figure S5**	Example of augmented spectra produced by EMSA	S5
**Figure S6**	Comparison of TGA-FTIR and ATR-FTIR spectra of pristine and environmentally weathered polypropylene	S6
**Figure S7**	“Limit of Classification” and model performance vs sample complexity	S6

Table S1 – List of polymers measured to create the TGA-FTIR library & training data. FTIR spectra that occurred within T_{max} ± 5°C were used for the Pearson-R library. Spectra that fell within the regions of interest (ROI) were used for machine-learning training data.

Polymer	Supplier(s)	Form	T_{max} (°C)	Region(s) of Interest (°C)
Polyethylene (PE)	Sigma	powder	475	450-550
	Polysciences Inc.	microparticle powder	470	420-520
	Kal-Polymers	pellet	470	430-530
Polypropylene (PP)	Polysciences Inc.	microparticle powder	420	350-500
	Kal-Polymers	pellet	455	390-500
Polystyrene (PS)	Sigma	powder	415	370-490
	Cospheric	microparticle powder	420	360-500
	Kal-Polymers	pellet	415	380-480
Polyethylene terephthalate (PET)	Sigma	powder	420	370-550
	Goonvean Fibers	fiber	430	390-520
	Kal-Polymers	pellet	440	390-520
Polyamide (PA)	Goonvean Fibers	fiber	450	390-510
	Magerial (PA 6)	powder	450	370-550
	Magerial (PA 6-6)	powder	450	390-520
Polyurethane (PUR)	Kal-Polymers	film	410	325-500
Polyvinyl Chloride (PVC)	Sigma	powder	295;450	280-520
	Magerial	powder	285;470	270-520
	Kal-Polymers	pellet	300;450	250-500
Polymethyl methacrylate (PMMA)	Sigma	powder	285;360	150-420
	Magerial	powder	380	300-480
	Cospheric	microparticle powder	285;365	150-450
Polyteyrafluouroethylene (PTFE)	Polysciences Inc.	microparticle powder	595	490-690
	Polysciences Inc.	microparticle powder	590	490-690
Polycarbonate (PC)	Magerial	Powder	500	420-620
	Nanochemazone	Powder	470	415-550
Cellulose	Sigma	Powder	330	290-450
	NRC-CRM	crystalline powder	290	270-390

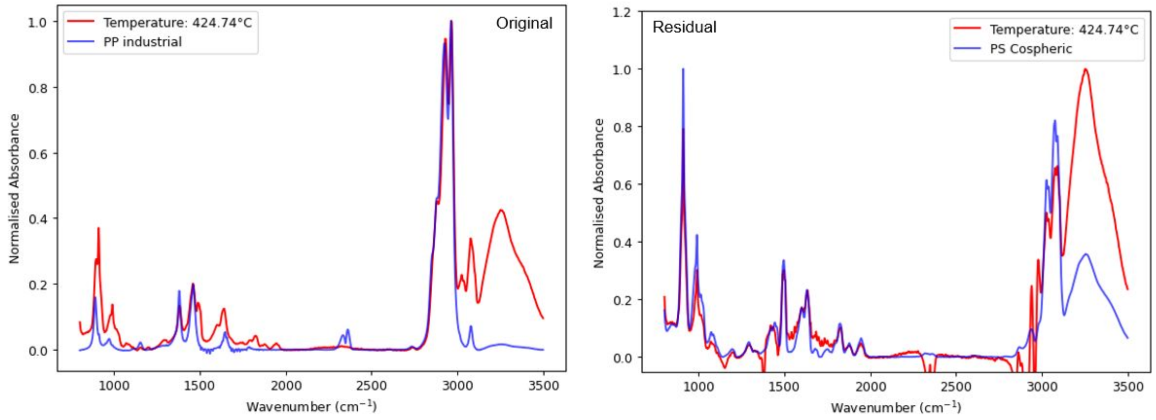

ID	r
polypropylene	0.889
polyvinyl chloride	0.737
polyethylene	0.6717

ID	r
polystyrene	0.752
polyvinyl chloride	0.371
polymethyl methacrylate	0.194

Figure S1 – Illustrating the residual matching process for identifying multiple polymers in a mixed spectrum. The overlay of the original data (red) is shown with the highest-scoring match (blue). The r values for the top three matches are displayed. The highest matching library spectrum is subtracted from the original data and matching is re-run on the residual. In this case, the residual correctly shows a high correlation for polystyrene, a component which was not identified in the original spectrum

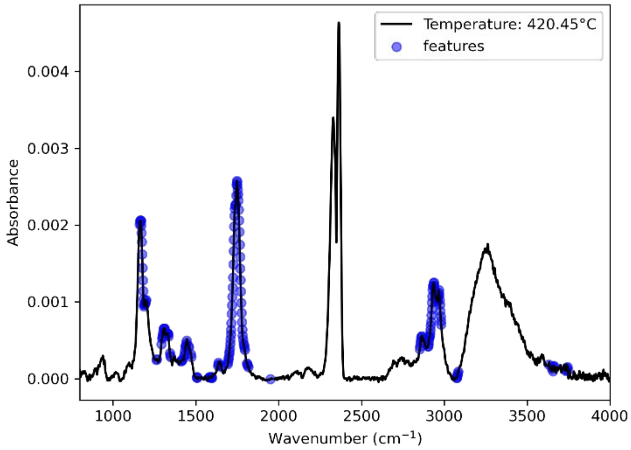

Figure S2 - Demonstration of feature selection. In this case, what were determined to be the 200 most relevant points are overlaid on this TGA-FTIR spectrum of a polymer mixture containing PE, PP, PET and PTFE.

Table S2 – Range of hyperparameter values tested during gridsearch cross-validation and the values used in the final models

Model	*Hyperparameter*	*Values tested*	*Value used*
Multilayer perceptron (MLP)	hidden layer sizes	5 – 50	20
	α	0.0001 – 10	0.01
Support vector classifier (SVC)	C	0.001 – 1000	0.1
	Kernel	linear, poly, rbf	Linear
k-nearest neighbor (kNN)	n neighbors	1 –10, 10 –100	5
	weights	uniform, distance	Uniform
Random Forest Classifier (RF)	n estimators	10 – 200	50
	max depth	2 – 5, None	None
	max features	0.1 – 1	0.1

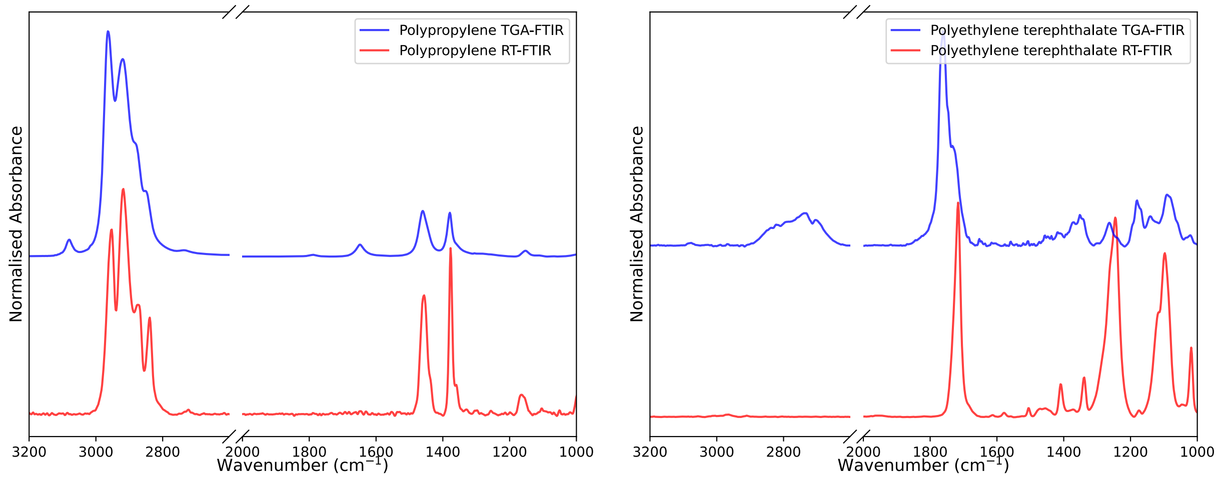

Figure S3 - Comparison of room temperature (RT-FTIR) and TGA-FTIR spectra for polypropylene and polyethylene terephthalate. The main spectral features, such as the peaks at 1460 and 1380 cm^{-1} remain consistent between both spectra of PP, but many peaks are shifted and radically altered in the case of PET.

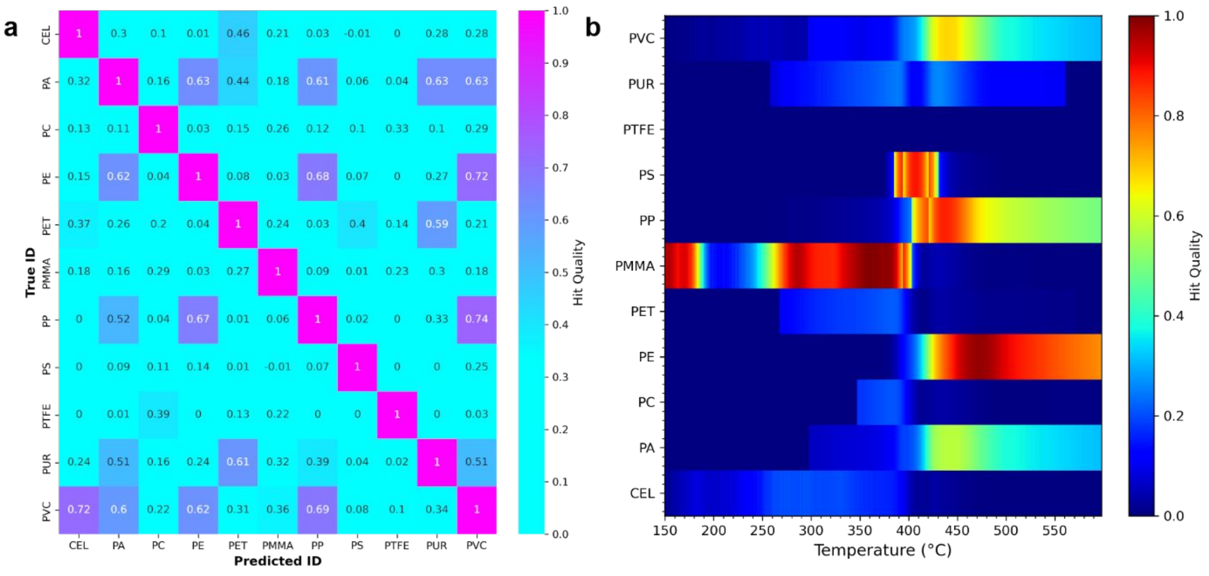

Figure S4 – a) A correlation matrix using the spectral matching algorithm, which includes 1st derivative matching and temperature penalization in contrast to the Pearson correlation matrix in Figure 2, along with b) the spectral matching results of the same PMMA, PS, PE and PP mixture that is displayed in Figure 3. The primary components are identified, but the false components PVC and PA nearly meet the threshold of hit quality (0.7) considered for a positive match. This becomes exasperated in samples with more complex mixtures or background matrices.

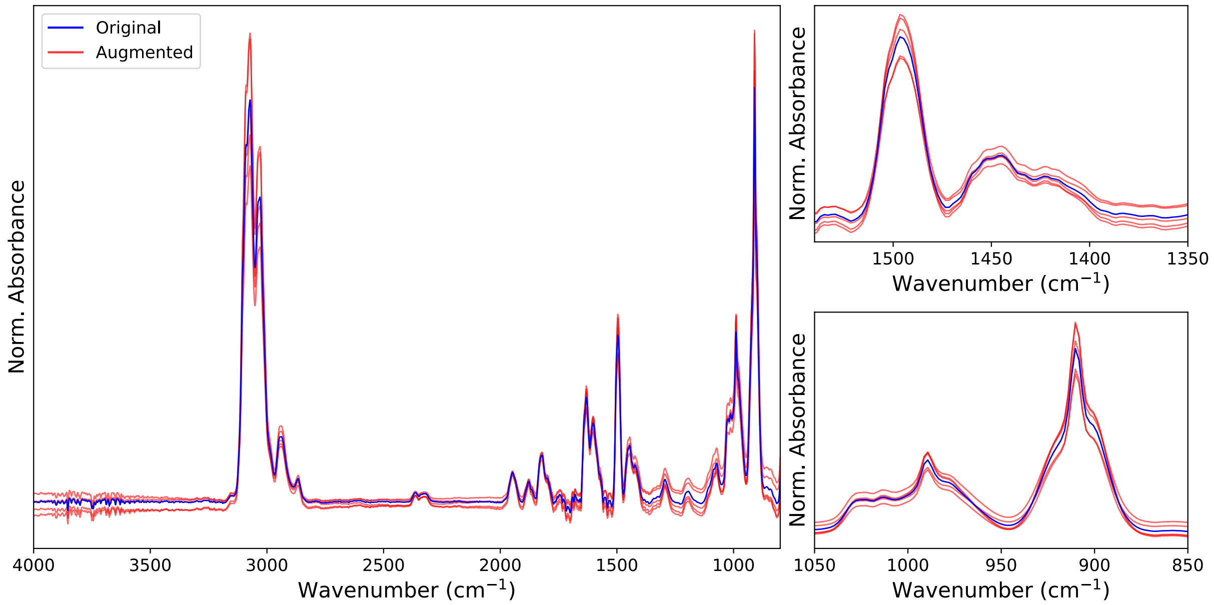

Figure S5 – Example of augmented spectra produced by Extended Multiplicative Scattering Augmentation (EMSA). The original spectrum is shown with 5 spectra which have been randomly generated.

The EMSA process was implemented as follows: For each class in the library a reference spectrum was generated by averaging a subsample (10%) of the spectra in this class. Each spectrum contributing to the reference spectrum is subjected to 2nd order extended multiplicative scattering correction (EMSC), which produces a set of coefficients. Augmented spectra are produced by applying EMSC in reverse on a random spectrum in the library from that class, using random coefficients bound by the standard deviation of the coefficients produced by the reference spectrum, and this process is repeated up to the desired number of times. An SVC model evaluated on the synthetic library showed an increase in F_1 score and Hamming accuracy from 0.90 to 0.92, and 0.84 to 0.87 respectively, demonstrating the data augmentation enhanced model performance.

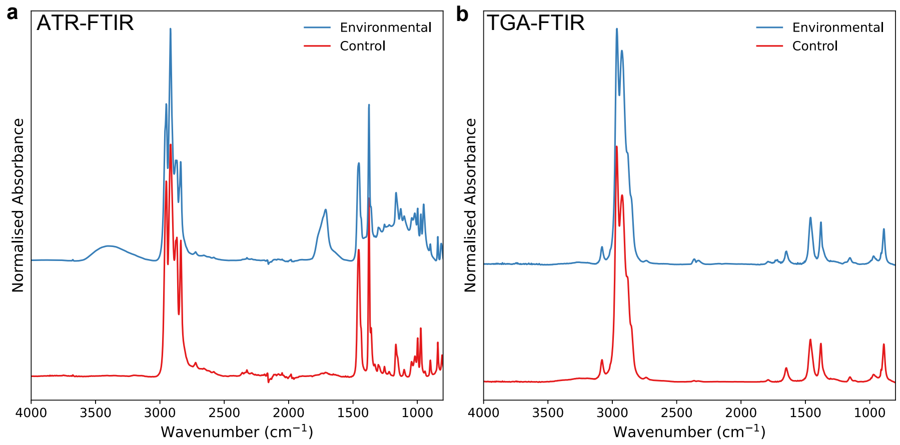

Figure S6 – A comparison of the a) TGA-FTIR spectra and b) ATR-FTIR spectra of a pristine piece of polypropylene and polypropylene recovered from a beach which has experienced environmental weathering. The ATR-FTIR spectrum shows the characteristic appearance of a broad OH band (3100 – 3600 cm^{-1}), and strong C=O band (~1750cm^{-1}), as well as changes to the fingerprint region and background. These differences can be characterized by a Pearson correlation coefficient of 0.885 between the two spectra. b) Meanwhile, the TGA-FTIR spectra only show minor differences in the C=O region, and the Pearson correlation between them is 0.997.

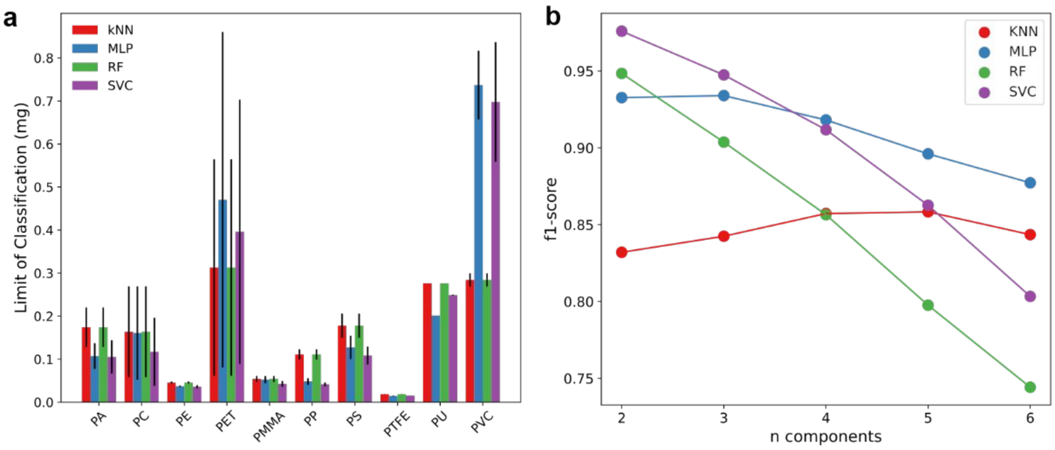

Figure S7 – a) The estimated limit of classification (LOC) of each polymer in the library; the minimum mass in a sample required for the classifier to correctly identify the presence of the polymer. In general, these correlate well with the model performance of each polymer, as seen in Figure 5. The LOCs of well-performing polymers, such as PE, PP (~0.04 mg) are in agreement with expectations of experimental LODs by TGA. This was prepared with synthetic data, mixing polymer samples with a blank sample with decreasing fractions until the model failed to identify the presence of the polymer, then applying this fraction of the original mass of the sample.

b) the relationship between model performance, exemplified by F_1 score, and the number of polymer components in each sample in a synthesized dataset. As the mixtures become more complex, the model performance decreases. This will likewise affect the LOD/LOC of evaluating the sample. The exception being with kNN, which had poor recall to start with. was prepared by evaluating the models on a synthetic dataset. The dataset was prepared by "mixing" data from n different randomized polymers into new synthetic thermograms, producing 500 TGA-FTIR datasets each containing n components.
